# The pathophysiology, diagnosis, and management of sepsis-associated disseminated intravascular coagulation

**DOI:** 10.1186/s40560-023-00672-5

**Published:** 2023-05-23

**Authors:** Toshiaki Iba, Julie Helms, Jean Marie Connors, Jerrold H. Levy

**Affiliations:** 1 Department of Emergency and Disaster Medicine, Juntendo University Graduate School of Medicine, 2-1-1 Hongo Bunkyo-Ku, Tokyo, 113-8421 Japan; 2 Université de Strasbourg (UNISTRA), Faculté de 1Médecine, Hôpitaux Universitaires de Strasbourg, Service de Médecine Intensive-Réanimation, Nouvel Hôpital Civil, 1, place de l’Hôpital, 67091 Strasbourg Cedex, France; 3 INSERM (French National Institute of Health and Medical Research), UMR 1260, Regenerative Nanomedicine (RNM), FMTS, Strasbourg, France; 4 Hematology Division Brigham and Women’s Hospital, Harvard Medical School, Boston, MA USA; 5 Department of Anesthesiology, Critical Care, and Surgery, Duke University School of Medicine, Durham, NC USA

**Keywords:** Sepsis, Disseminated intravascular coagulation, Coagulopathy, Endothelial cell, Antithrombin, Thrombomodulin

## Abstract

**Background:**

The International Society on Thrombosis and Haemostasis (ISTH) released overt disseminated intravascular coagulation (DIC) diagnostic criteria in 2001. Since then, DIC has been understood as the end-stage consumptive coagulopathy and not the therapeutic target. However, DIC is not merely a decompensated coagulation disorder, but also includes early stages with systemic activation in coagulation. Thus, the ISTH has recently released sepsis-induced coagulopathy (SIC) criteria that can diagnose compensated-phase of coagulopathy with readily available biomarkers.

**Main body:**

DIC is a laboratory-based diagnosis due to various critical conditions, although sepsis is the most common underlying disease. The pathophysiology of sepsis-associated DIC is multifactorial, and in addition to coagulation activation with suppressed fibrinolysis, multiple inflammatory responses are initiated by activated leukocytes, platelets, and vascular endothelial cells as part of thromboinflammation. Although overt DIC diagnostic criteria were established by ISTH to diagnose the advanced stage of DIC, additional criteria that can detect an earlier stage of DIC were needed for potential therapeutic considerations. Accordingly, the ISTH introduced SIC criteria in 2019 that are easy to use and require only platelet count, prothrombin time-international normalized ratio, and Sequential Organ Failure Assessment Score. SIC score can be used to evaluate disease severity and determine the timing of potential therapeutic interventions. One of the major disadvantages in treating sepsis-associated DIC is the lack of availability of specific therapeutic approaches beyond treating the underlying infection. Clinical trials to date have failed because included patients who were not coagulopathic. Nevertheless, in addition to infection control, anticoagulant therapy will be the choice for sepsis-associated DIC. Therefore, the efficacy of heparin, antithrombin, and recombinant thrombomodulin has to be proven in future clinical studies.

**Conclusion:**

It is necessary to develop a novel therapeutic strategy against sepsis-associated DIC and improve the outcomes. Consequently, we recommend screening and monitoring DIC using SIC scoring system.

## Introduction

**Fig. 1:**
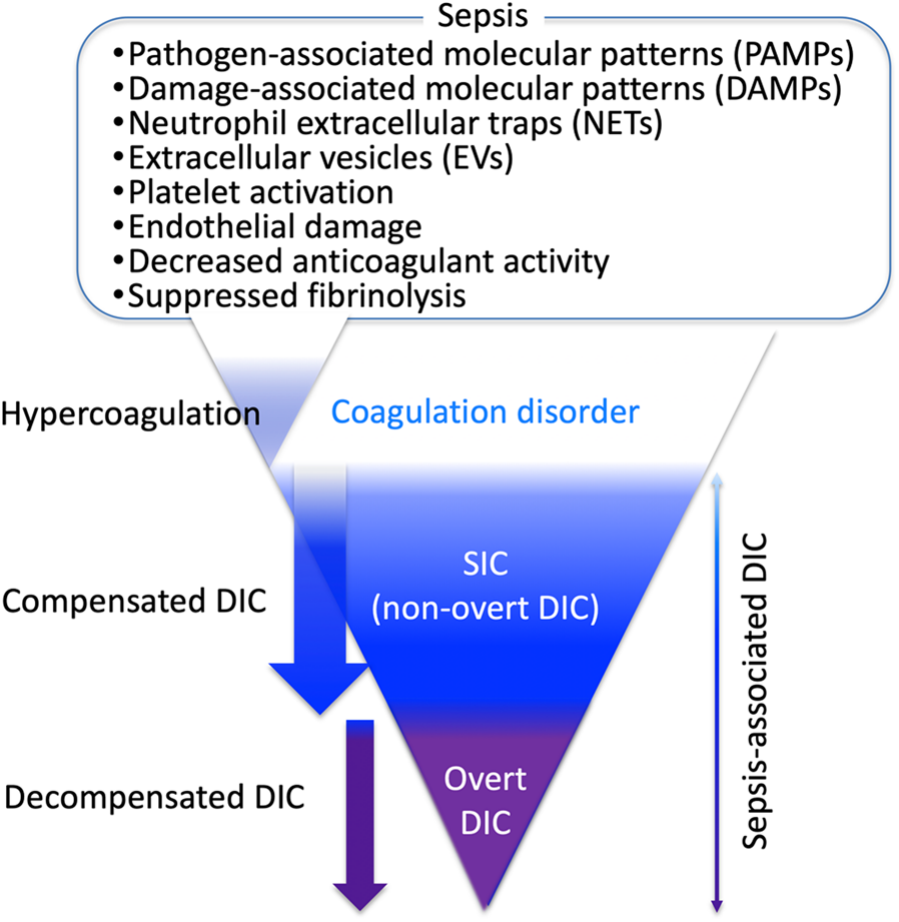
The initiation and progression of thromboinflammation in sepsis. The inflammation and host responses to infection induce coagulation disorder in sepsis. Although coagulation disorder is a part of host defense, excess coagulation disorder can be harmful to the host. Disseminated intravascular coagulation (DIC) is a systemic coagulation disorder that can cause the disturbance of tissue malcirculation and subsequent organ dysfunction. Sepsis-induced coagulopathy (SIC) is an early phase of DIC and a potential target of anticoagulant therapy. The advanced stage of DIC is defined as overt DIC and potentially the target of supplementation therapy

Hemostasis, a fundamental host defense mechanism against various pathophysiologic insults, clinically presents as macro- and microthrombosis in acute injury and critically ill patients. Although the hemostatic response is beneficial in some conditions, excess thromboinflammation also induces tissue malcirculation and causes subsequent organ dysfunction [1]. Systemic hypercoagulation with or without consumptive coagulopathy frequently occurs following major tissue injury, and based on laboratory-based disseminated intravascular coagulation (DIC) diagnostic criteria, patients are clinically diagnosed as having DIC [2]. The pathophysiology, phenotypic expression, and treatments of DIC vary considerably based on the underlying causative diseases [3, 4]. However, sepsis is the most frequent and life-threatening cause of DIC and commonly presents as a thrombotic type DIC [5, 6]. This type of DIC is often complicated by organ dysfunction, with hemorrhagic events occurring less commonly [7]. Coagulation status changes dynamically based on the progression of the underlying disease. Sepsis-associated DIC is a dynamic condition that starts from coagulation disorder, can advance to sepsis-induced coagulopathy (SIC), and finally to decompensated coagulation disorder (overt DIC) (Fig. 1). Anticoagulation is expected for the compensated DIC, and the decompensated stage may require additional therapeutic modalities with supplementation therapy [8, 9]. Recent advances in sepsis-associated DIC management include the development of early diagnostic criteria based on readily available clinical information and the administration of potentially effective anticoagulants [4, 10]. This review will focus on advances in sepsis-associated DIC diagnosis, management, and future perspectives.

## Pathophysiology of sepsis-associated DIC

**Fig. 2:**
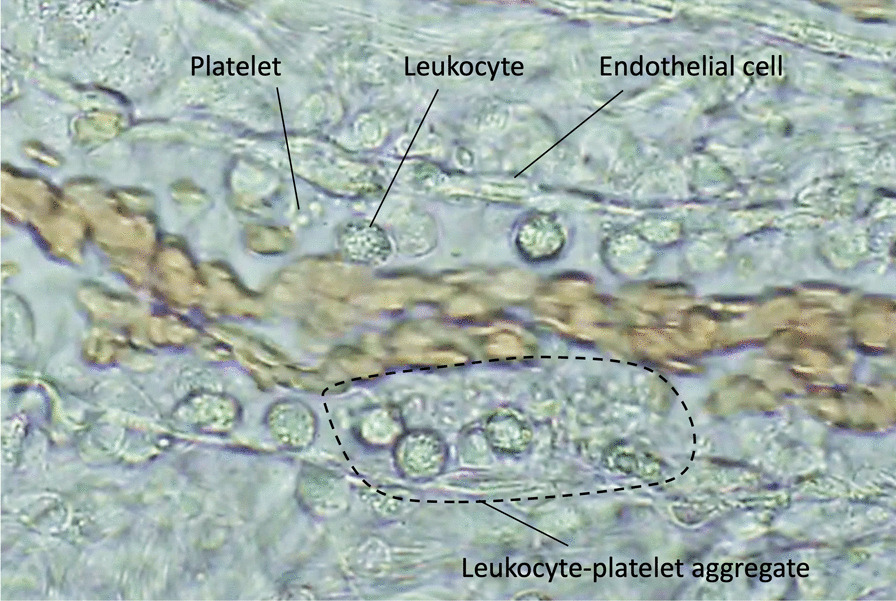
Microthrombosis in the microcirculation in sepsis model of rat. Rat mesenteric microcirculation was observed under the intravital microscope after lipopolysaccharide injection. Round stiff leukocytes adhered to the unsmooth and swelled endothelial cells. Some leukocytes transmigrated to the extravascular space. Aggregated platelets stick to the leukocytes and endothelium and form leukocyte–platelet aggregation. Along with these changes, the blood flow gradually decreased

**Fig. 3:**
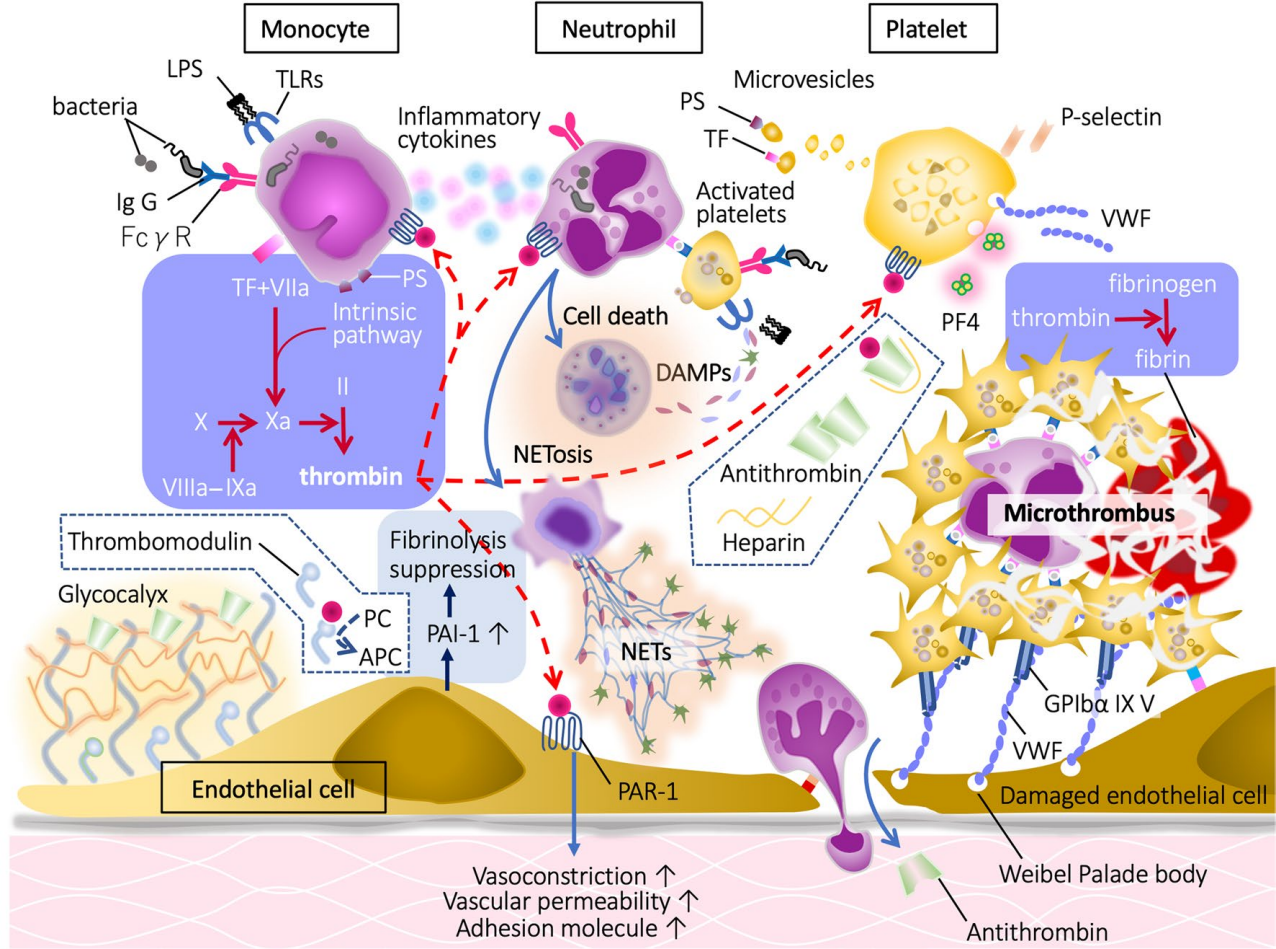
Pathophysiology of sepsis-associated coagulation disorder. Monocytes, neutrophils, and platelets express various receptors such as Toll-like receptors (TLRs), Fcγ receptor (FcγR), and protease-activated receptor (PAR)-1, and induce inflammatory and procoagulant responses in these cells. Activated monocytes express tissue factor (TF) and phosphatidylserine (PS) on the surface, which initiate coagulation cascades. At the same time, monocytes release proinflammatory cytokines and stimulate neutrophils. Activated neutrophils release damage-associated molecular patterns (DAMPs) after cell death, and expel extracellular traps (NETs) that facilitate inflammation through binding to TLRs. Platelets release procoagulant microvesicles and prothrombotic substances such as von Willebrand factor (VWF) and platelet factor 4 (PF4). VWF helps platelet aggregation, and PF4 neutralizes heparan sulfate of the glycocalyx. Vascular endothelial cells lose anti-thrombogenicity by decreasing the production of nitric oxide (NO) and prostaglandin I_2_ (PG I_2_), and by increasing the production of plasminogen activator inhibitor 1 (PAI-1). Thrombomodulin which converts protein C (PC) to its activated form activated protein C (APC) is released from endothelial cells, and antithrombin is extravasated with increased vascular permeability. *LPS* lipopolysaccharide, *Ig G* immunoglobulin G, *GP* glycoprotein

Monocytes/macrophages are the first-line responders to the invaded pathogens and phagocyte pathogens that detect bacteria by sensing specific molecular patterns and elicit proinflammatory and procoagulant reactions to entrap and localize them [11]. As pattern-recognizing receptors, monocytes/macrophages express Toll-like receptors (TLRs), Fcγ-receptors, and G-protein-coupled receptors that detect the pathogen-associated molecular patterns (PAMPs), subsequently integrating the innate immune system as well as the coagulation system [12]. TLRs not only react to PAMPs, but also transduce the binding signals of host-derived stress molecules, namely damage-associated molecular patterns (DAMPs), thereby initiating a vicious cycle of inflammation and coagulation [13]. After the subsequent multi-step signal transduction, monocytes/macrophages produce proinflammatory cytokines and chemokines that activate neutrophils and upregulate the expression of tissue factor (TF) and phosphatidylserine (PS) on the cellar membrane [14]. TF and PS initiate extrinsic and intrinsic coagulation cascades and facilitate prothrombotic reactions. Activated neutrophils kill pathogens by using proteases, reactive oxygen species, releasing neutrophil extracellular traps (NETs), and further propagating inflammation by cell death mechanisms [15]. Pyroptosis, necroptosis, NETosis, and necrosis are the representative cell death styles that increase inflammation and coagulation by releasing DAMPs and other cytotoxic substances [16]. Platelets also participate in the host defense by developing thrombus formation via expressing adhesion molecules, releasing granule components such as platelet factor 4 (PF4), von Willebrand factor (VWF) [17], and procoagulant microvesicles [18]. These responses lead to intravascular microthrombus formation (immunothrombosis) to protect the host against infection (Fig. 2). Among the coagulation factors, thrombin is a critical mediator that regulates inflammation and coagulation [19]. Monocyte, neutrophil, platelet, and endothelial cells also express protease-activated receptor-1 (PAR-1), and thrombin upregulates proinflammatory and procoagulant reactions by binding to PAR-1 [19, 20]. Following sepsis-induced acute inflammatory responses, vascular endothelial cells lose their antithrombotic properties by factors that include decreased nitric oxide/prostaglandin I_2_ production, and disruption of the glycocalyx. Thrombomodulin is proteolytically cleaved and released from the endothelial surface. In addition, the production of plasminogen activator inhibitor 1 (PAI-1) is upregulated, and fibrinolytic suppression becomes evident. This suppression in fibrinolysis is the critical feature of coagulation disorder in sepsis (fibrinolytic shutdown). Damaged endothelial cells release thrombogenic molecules, including VWF and adhesion molecules, and stimulate platelet adhesion and aggregation [21]. Furthermore, physiological anticoagulant factors such as antithrombin and protein C are extravasated with increased vascular permeability [22]. These events facilitate widespread microthrombus formation during sepsis (Fig. 3).

## Diagnosis of sepsis-associated DIC

DIC is defined as the systemic activation of coagulation and not the consumptive coagulopathy [23], but many physicians consider DIC as a decompensated coagulation disorder, potentially due to the 2001 definition of overt DIC criteria by the International Society on Thrombosis and Haemostasis (ISTH) that is defined as thrombocytopenia, significant prolongation of prothrombin time (PT), moderate or strong increase of fibrin related markers, and/or decreased fibrinogen level. It has not been well-known, but the ISTH has also defined non-overt DIC diagnostic criteria determined by sensitive and specific molecular markers that include antithrombin, protein C, or thrombin–antithrombin (TAT) complexes [23]. However, many patients developed overt DIC independently of non-overt DIC. In addition, since the molecular markers were not commonly used clinically, they were not suitable to use for diagnosing non-overt DIC.

Despite the presence of these classical definitions and criteria, the establishment of new DIC diagnostic criteria was demanded to identify the potential therapeutic target. Consequently, the Japanese Society for Acute Medicine (JAAM) released its compensated DIC criteria by using readily available laboratory tests and clinical data in 2006 [24]. However, after the update of the sepsis definition to Sepsis-3 [25, 26], the JAAM DIC criteria became outdated because the JAAM DIC criteria included the systemic inflammatory response syndrome (SIRS) score. SIC criteria were then introduced in 2017 to diagnose the early onset DIC in sepsis [27]. SIC criteria are defined by the presence of sepsis (infection with organ failure [increased Sequential Organ Failure Assessment (SOFA) score]) and coagulation disorder (decreased platelet count and prolonged PT-international normalized ratio [INR]) [27] (Table 1). The measurement of the platelet count and PT-INR are routine clinical tests, and the SOFA score is an easily calculated clinical score used to evaluate the severity of sepsis [28]. Screening for DIC in septic patients is important based on a large Japanese study reporting efficacy of anticoagulant therapy for coagulopathic septic patients with a high risk of death [29]. After additional clinical reports [30, 31], SIC diagnostic criteria were approved by ISTH DIC Scientific Standardization Committee (SSC) [32, 33]. The clinically relevant importance of the SIC criteria is its simplicity and ability to facilitate the timing of anticoagulant therapy [34]. The JAAM DIC criteria are used popularly for the diagnosis of sepsis-associated DIC in Japan. To mention the superiority of SIC scoring over JAAM DIC scoring, we have to show a better treatment effect based on the SIC diagnosis than that based on the JAAM DIC diagnosis. However, such a study has not been done yet. But, SIC criteria are simpler than JAAM DIC criteria, less costly, and therefore, more suitable for monitoring. In addition, SIC has been shown to directly continue to overt DIC with disease progression.

**Table 1:** ISTH overt DIC, JAAM DIC, and SIC scoring systems

Item	Score	ISTH overt DIC	JAAM DIC	ISTH SIC
		Range	Range	Range
Platelet count (× 10^9^/L)	3	−	< 80 or ≧ 50% decrease within 24 h	−
	2	< 50	−	< 100
	1	≧ 50, < 100	120 > , 80 ≦ or ≧ 30% decrease within 24 h	≧ 100, < 150
FDP (D-dimer)	3	strong increase	≧ 25 μg/mL (use convert chart)	−
	2	moderate increase	−	−
	1	−	≧ 10, < 25 μg/mL (use convert chart)	−
Prothrombin time	2	≧ 6 s	−	> 1.4
	1	≧ 3 s, < 6 s	≧ 1.2 (PT ratio)	> 1.2, ≦ 1.4 (PT-INR)
Fibrinogen (g/mL)	1	< 100	−	−
SIRS score	1	−	> 3	−
SOFA score	2	−	−	≧2
	1	−	−	1
Total score for DIC or SIC		≧ 5	≧ 4	≧ 4

Total SOFA score is the sum of 4 items (respiratory SOFA, cardiovascular SOFA, hepatic SOFA, and renal SOFA)

*ISTH* International Society on Thrombosis and Haemostasis, *DIC* disseminated intravascular coagulation, *JAAM* Japanese Society for Acute Medicine, *SIC* sepsis-induced coagulopathy, *SIRS* systemic inflammatory response syndrome, *SOFA* Sequential Organ Failure Assessment

SIC criteria were specifically designed for sepsis-associated DIC. Likewise, establishing underlying disease-specific considerations may allow us to develop simple and better criteria; we think this approach should be expanded to other areas of DIC, i.e., hematologic DIC and cancer-induced DIC.

## The prevalence and mortality of sepsis-associated DIC

In sepsis, the prevalence of DIC varies depending on the target cohorts and diagnostic criteria, but the mortality is always higher in DIC patients compared to non-DIC patients. Gando et al. [35] examined the incidence and the mortality of overt DIC and JAAM DIC in septic patients (Sepsis-1) and reported that they were 18.1% vs. 46.8%, and 38.1% vs. 38.4%, respectively. Another retrospective cohort study examined the prevalence and the mortality of overt DIC, JAAM DIC, and SIC in 1892 patients with sepsis (Sepsis-1) and reported that they were 29.3% and 38.4%, 61.4% and 33.9%, and 60.8% and 32.5%, respectively [36]. Data from a Sepsis-3 cohort of 296 subjects reported the prevalence of overt DIC, modified-JAAM DIC (SIRS score was replaced with antithrombin activity), and SIC were 22.6%, 43.2%, and 56.1%, and 28-day mortalities were 55.2%, 47.7%, and 44.0%, respectively [37]. These data suggest that the prevalence of DIC differs based on definitions, but the mortality of sepsis-associated DIC exceeded 30% regardless of the diagnostic criteria. The primary objective of diagnosing DIC is not to predict outcomes but to decide the timing of initiating intervention because DIC patients have higher mortality compared to non-DIC patients [38]. Of note, recent studies support the idea that anticoagulant therapy is more effective for patients with coagulopathy/DIC and high disease severity [38, 39]. Further, the JAAM DIC and SIC scores reflect disease severity, and the relationship between the scores and mortality was reported [24, 27]. Lu et al. [40] evaluated performance of SIC scoring in 9432 sepsis patients and reported that 28-day mortality was 34% in SIC patients compared to 25% in non-SIC patients. They also reported that the presence of SIC was an independent risk factor for 28-day mortality with an adjusted odds ratio of 1.52 (95% confidence interval [CI]: 1.39 to 1.67).

## The performance of SIC scoring system

The usefulness of diagnostic criteria has been examined by evaluating their performance to discriminate the patients with a high risk of death. Wang et al. [37] compared the performance of various DIC diagnostic criteria including SIC, modified-JAAM DIC, simplified Japanese Society on Thrombosis and Hemostasis DIC, and overt DIC criteria in predicting 28-day mortality and reported that the area under the curves (AUC) did not differ among the diagnostic criteria (AUC: between 0.730 and 0.763); nevertheless, it is impactful that SIC is the simplest criterion among them and the odds ratio to death was highest and 5.218 (95% CI: 2.878 to 9.459, *P* < 0.001). As a screening test like SIC scoring, the expected performance is the capability to deny death when SIC is negative. In this sense, the negative predictive value for 28-day mortality was also highest in SIC and 86.9%. Similar results were confirmed in multiple studies, and the SIC score was reported to be associated with the severity of diseases [40–49].

The primary objective of diagnosing DIC was not to identify septic patients with a high risk of death but to identify patients who may benefit from anticoagulant therapy. Zou et al. [47] evaluated 6646 septic patients retrospectively and reported the association between heparin administration and lower mortality only in patients with SIC (hazard ratio [HR]: 0.74, 95% CI: 0.65 to 0.85). In contrast, the association was not observed in patients without SIC (HR: 0.95, 95% CI: 0.64 to 1.40). Peng et al. [48] also examined the effect of heparin on septic patients in their retrospective study and concluded that patients with SIC could be good candidates for heparin therapy. In their study, most of the overt DIC patients were diagnosed with SIC, and most overt DIC patients had received a prior diagnosis of SIC [30]. Therefore, SIC precedes overt DIC, and patients progress from SIC to overt DIC with disease progression.

One limitation of SIC criteria is the risk of misdiagnosing diseases that mimic DIC, which may not be small as only two coagulation biomarkers are included. For example, it is not easy to differentiate the thrombotic microangiopathic (TMA) disorders such as thrombotic thrombocytopenic purpura (TTP) [49] and atypical hemolytic uremic syndrome (aHUS) [50], and heparin-induced thrombocytopenia (HIT) from SIC [51]. To support the accurate diagnosis, the ISTH DIC SSC provides a flow chart that aids with systematic differential diagnosis [52]. Although the prolongation of PT-INR is mild, TMA disorders commonly have significant thrombocytopenia and achieve sufficient scores. The decrease in platelet count is more prominent, and the changes in PT are milder in TTP, aHUS, and HIT, and the balance of platelet count and PT can be useful for differentiation. In addition, antithrombin activity, a biomarker of endothelial damage, is significantly decreased in SIC and DIC [53, 54], but it is usually maintained in the TMA and can be helpful for discrimination [55, 56]. Antithrombin level is known to decrease in the early phase of DIC. Jackson Chornenki et al. [57] reported that platelet count and PT-INR in combination with antithrombin activity could identify early-phase DIC in septic patients. Although the incidences are not high, the differential diagnosis of these DIC-mimicking diseases is critical because the mortality is considerably high if treated inappropriately.

## How should we treat sepsis-associated DIC?

**Fig. 4:**
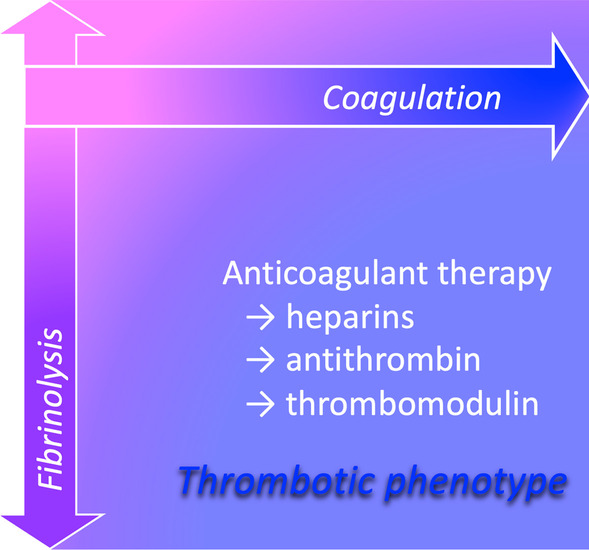
The therapeutic target of anticoagulant. The most popular anticoagulants worldwide are heparins, meanwhile, antithrombin and recombinant thrombomodulin are popularly used for disseminated intravascular coagulation (DIC) in Japan. For more than a decade, it has been reported that anticoagulant therapy is effective only for patients with coagulation disorder, and recent studies have shown that sepsis-associated DIC with high disease severity is the optimal target

The treatment of underlying disease is essential in DIC, but for sepsis-associated DIC, additional treatment for ongoing tissue poor perfusion is critical in clinical management. However, little evidence supports the efficacy of anticoagulant therapy in addition to antibiotics and source control. Despite studies of several anticoagulants in large-scale randomized controlled trials (RCTs), no current anticoagulant has yet demonstrated efficacy [61, 63]. Potential reasons include the variability of clinical presentation in sepsis [61], but as previously described, anticoagulant therapy should be considered only for patients with a coagulation disorder, as best noted the optimal target is sepsis-associated DIC with high disease severity [38, 39] (Fig. 4).

### Heparin

Heparin is commonly used in septic patients for multiple reasons. However, no large randomized controlled study has examined heparin alone for sepsis-associated DIC. A recent meta-analysis reported that unfractionated heparin could reduce the mortality of sepsis, especially in patients with high severity [62]. However, the study population was not restricted to patients with coagulopathy/DIC. Another systematic review of heparin in DIC [63] was limited by including studies in which three out of eight studies used heparin as the control. Although heparin is strongly recommended for COVID-19 [64], its efficacy for sepsis-associated DIC is still unclear. A Japanese RCT compared the efficacy and safety of recombinant thrombomodulin to heparin in DIC-associated with hematologic malignancy or infection. In subgroup analysis, 80 patients with sepsis-associated DIC were subjected to the analyses. The results showed DIC resolution rates in recombinant thrombomodulin group and heparin group were 67.5% and 55.6%, and 28-day mortality rates were 21.4% and 31.6%, respectively [65].

### Recombinant thrombomodulin

Recombinant thrombomodulin was developed and approved for clinical use in Japan in 2008 for the treatment of DIC [66]. As described before, Aikawa et al. [65] performed a subgroup analysis on 80 sepsis-associated DIC patients from a Phase 3 trial [67] and reported better performance of recombinant thrombomodulin. The subsequent SCARLET trial reported in 2019 enrolled septic patients with coagulopathy and organ dysfunction (cardiovascular and/or respiratory failure, platelet count 30 to 150 × 10^9^/L or > 30%-decrease within 24 h, and PT-INR > 1.4 [SIC score > 4]) [68]. Although the difference was not statistically significant in the intention-to-treat population, a trend of better 28-day survival was observed in the subgroup that fulfilled the entry criteria at baseline (difference: 5.40%, 95% CI: -1.68% to 12.48%). In this trial, a significant inter-nations difference was noted, and a post hoc analysis revealed absolute risk reductions of 8.3% in the French population in contrast to 1.1% in the rest of the world; the greater effect in the French cohort was thought to be due to a higher rate of sustained coagulopathy and a lower rate of heparin use [69]. It is noteworthy that all the RCTs showed trends toward better outcomes in the treatment group, and a meta-analysis of RCTs showed a significant improvement in mortality [70]. Consequently, recombinant thrombomodulin is recommended for sepsis-associated DIC in Japanese sepsis guidelines [71].

### Antithrombin

The largest RCT that examined the effect of antithrombin was performed in severe sepsis but not in sepsis-associated DIC patients [58]. However, Kienast et al. [72] performed a post hoc analysis in 563 patients (antithrombin group: n = 286, and placebo group: n = 277) who had DIC and did not receive concomitant heparin, and reported an absolute reduction of 14.6% in 28-day mortality in the antithrombin group (*P* = 0.02), whereas no such effect was seen in patients without DIC. Antithrombin is used for the treatment of DIC in Japan, and that is the recommendation of Japanese sepsis guidelines [71]. Japanese recommendations are based on data by Tagami et al. [73], who performed propensity score-matched analysis using a nationwide administrative database consisting of 9075 pneumonia patients with sepsis-associated DIC (antithrombin: n = 2663 and control: n = 6412) and created a matched cohort of 2194 pairs. The result reported a mortality difference between the groups (antithrombin: 40.6% vs. control: 44.2%, *P* = 0.02). Furthermore, multiple logistic regression analyses showed an association between antithrombin use and 28-day mortality (odds ratio [OR] 0.85, 95% CI: 0.75 to 0.97). In addition to the plasma-derived product, recombinant antithrombin (antithrombin-γ) was approved in Japan in 2015, and better performance in terms of improvements in DIC and SOFA scores was reported in small studies [74, 75]. Consistent with these reports, Japanese Sepsis guidelines recommend the use of antithrombin and recombinant thrombomodulin for sepsis-associated DIC [71].

Recently, a potentially beneficial effect of the combination therapy of antithrombin with recombinant thrombomodulin has been reported, especially in severe cases [76]. The effect of this combination therapy should be examined in a future trial.

## Future perspectives

The mortality associated with sepsis as well as sepsis-associated DIC remains high and exceeds 30%. Accumulated evidence suggests that anticoagulant therapy is effective for critically ill patients with coagulation disorders. Sepsis is a systemic response that acutely causes multiorgan dysfunction, and is treatable with antibiotics and source control in its early phase. However, the condition gets worse once it is complicated by coagulation disorder. Therefore, it is important to screen and monitor coagulation changes in septic patients routinely with readily available methods. This review focuses on sepsis-associated DIC rather than covering all types of DIC. The SIC scoring system is specifically designed for diagnosing sepsis-associated DIC in its early phase and is simple and easy to calculate, making it useful for DIC screening and monitoring purposes in the sepsis population. Such screening and monitoring have been performed in Japan using the JAAM DIC criteria. However, since the calculation of the SIRS score was no longer used in the diagnosis of sepsis, an update is necessary. SIC was introduced to Japan by the Japanese Sepsis Guideline Working Group in 2019 as alternative diagnostic criteria [77], and it will be included in the next version of the Japanese sepsis guidelines.

As for the treatment of SIC, heparins, antithrombin, and recombinant thrombomodulin are the current choices for sepsis-associated DIC. So far, none of them has robust evidence for efficacy. There are some obstacles to designing clinical trials that prove the effectiveness of these anticoagulants. First, the endpoint has been set as mortality difference; however, there are many factors that affect the patient’s outcome, and a more sophisticated endpoint that reflects the treatment effect of anticoagulation is warranted. Second, determining the optimal dose, duration, and termination is not easy. Since activation in coagulation is a part of host defense mechanisms, the selection of targets and the choice of agents are also important. Since anticoagulation has increasingly attracted attention [4, 8, 10], it is necessary to set up international collaborative studies that adopt proper endpoints.

## Conclusion

DIC is a common complication in sepsis. Although the mortality of sepsis-associated DIC is high, early detection and timely intervention are important to improve outcomes. DIC is a laboratory-based diagnosis, and the ISTH DIC SSC introduced SIC criteria which are simple, easy to calculate, and suitable for repeated evaluation to identify septic patients with a risk of progressing to overt DIC and death. Despite the advances in research on the pathophysiology, therapeutic agents for improving sepsis outcomes beyond antimicrobial treatment have never been recommended in most countries. Although recombinant thrombomodulin and antithrombin are approved by Japanese guidelines, further prospective international collaborative studies are needed.

## Data Availability

Not applicable.

## Abbreviations

DIC: Disseminated intravascular coagulation
ISTH: International Society on Thrombosis and Haemostasis
SIC: Sepsis-induced coagulopathy
TLRs: Toll-like receptors
PAMPs: Pathogen-associated molecular patterns
DAMPs: Damage-associated molecular patterns
TF: Tissue factor
PS: Phosphatidylserine
NETs: Neutrophil extracellular traps
PF4: Platelet factor 4
VWF: Von Willebrand factor
PAR-1: Protease-activated receptor-1
PAI-1: Plasminogen activator inhibitor 1
JAAM: Japanese Society for Acute Medicine
SIRS: Systemic inflammatory response syndrome
PT: Prothrombin time
INR: International normalization ratio
SOFA: Sequential Organ Failure Assessment
SSC: Scientific Standardization Committee
AUC: Area under the curves
HR: Hazard ratio
TTP: Thrombocytopenic purpura
aHUS: Atypical hemolytic uremic syndrome
HIT: Heparin-induced thrombocytopenia
RCT: Randomized controlled trials
OR: Odds ratio
